# Tailored therapy in patients treated with fluoropyrimidines: focus on the role of dihydropyrimidine dehydrogenase

**DOI:** 10.20517/cdr.2018.006

**Published:** 2019-09-19

**Authors:** Filippo Merloni, Nicoletta Ranallo, Laura Scortichini, Riccardo Giampieri, Rossana Berardi

**Affiliations:** ^1^Scuola di Specializzazione in Oncologia, Università Politecnica delle Marche, Ancona 60121, Italy.; ^2^Clinica Oncologica, Università Politecnica delle Marche, AOU Ospedali Riuniti, Ancona 60126, Italy.

**Keywords:** 5-fluorouracil, fluoropyrimidines, chemotherapy, dihydropyrimidine dehydrogenase, dihydropyrimidine dehydrogenase, *DPYD*, CPIC, polymorphisms

## Abstract

Fluoropyrimidines are widely used in the treatment of solid tumors, mainly gastrointestinal, head and neck and breast cancer. Dihydropyrimidine dehydrogenase (DPD) is the rate-limiting enzyme for catabolism of 5-FU and it is encoded by *DPYD* gene. To date, many known polymorphisms cause DPD deficiency and subsequent increase of 5-FU toxicity. In addition, reduced inactivation of 5-FU could lead to increased 5-FU intracellular concentration and augmented efficacy of this drugs. Therefore DPD expression, particularly intratumoral, has been investigated as predictive and prognostic marker in 5-FU treated patients. There also seems to be a tendency to support the correlation between DPD expression and response/survival in patients treated with fluoropyrimidine even if definitive conclusions cannot be drawn considering that some studies are conflicting. Therefore, the debate on intratumoral DPD expression as a potential predictor and prognostic marker in patients treated with fluoropyrimidines is still open. Four DPD-polymorphisms are the most relevant for their frequency in population and clinical relevance. Many studies demonstrate that treating a carrier of one of these polymorphisms with a full dose of fluoropyrimidine can expose patient to a severe, even life-threatening, toxicity. Severe toxicity is reduced if this kind of patients received a dose-adjustment after being genotyped. CPIC (Clinical Pharmacogenetics Implementation Consortium) is an International Consortium creating guidelines for facilitating use of pharmacogenetic tests for patient care and helps clinicians ensuring a safer drug delivery to the patient. Using predictive DPD deficiency tests in patients receiving 5FU-based chemotherapy, in particular for colorectal cancer, has proven to be a cost-effective strategy.

## Introduction

Fluoropyrimidines (FU) are the most prescribed anticancer drugs for the treatment of solid cancers, in particular breast, colorectal, head and neck, pancreas and gastric cancers^[1,2]^. The most common side effects are represented by emesis, bone marrow suppression, diarrhea, mucositis, fatigue and hand-foot syndrome. Fluoropyrimidines cause severe toxicities in 10% to 40% of patients and deaths in 0.2% to 0.8%^[3]^.

The correct management of fluoropyrimidine toxicity consists in the temporary suspension or interruption of treatment^[4-7]^. The metabolic pathway of 5-fluorouracil depends on the activity of many intracellular enzymes including dihydropyrimidine dehydrogenase (DPD)^[1]^.

DPD expression varies throughout several tissues and exerts his activity predominantly in liver, peripheral blood mononuclear cells, tumor and inflammatory tissues. Genotype can explain high evident interindividual variability of DPD levels and a circadian rhythm of expression of this enzyme is described^[8,9]^.

On top of that, differences in DPD activity have been reported among different ethnic groups^[10-12]^. Patients who receive 5-FU-based chemotherapy usually eliminate over 80% of this drug by converting it into its inactive metabolite 5,6-dihydro-5-fluorouracil^[4,13,14]^, owing to levels of DPD within the normal range. In patients showing DPD deficiency, decreased catabolism of 5-FU is observed and thus, increased risk of toxicity^[15]^.

In addition to a major risk of toxicity, reduced inactivation of 5-FU, could theoretically lead to increased 5-FU intracellular concentration and augmented efficacy of this chemotherapeutic agent. Therefore DPD expression, especially intratumoral, assessed by either enzymatic activity or mRNA expression, has been investigated as a predictive and prognostic marker in 5-FU treated patients, especially in those affected by colorectal cancer (CRC) given the prominent role of this chemotherapeutic agents in both metastatic and adjuvant setting.

In this review we will discuss the impact of 5-FU pharmacogenomics in the clinical outcome of patients treated with fluorouracil-based chemotherapy and in particular we will analyze the importance of polymorphisms of the enzyme DPD regarding the toxicity and efficacy of chemotherapy 5FU-based. We also want to underline how the prospective analysis of DPD mutations has a good cost-effectiveness ratio.

## Fluorouracil metabolism and DPD

### Fluoropyrimidines include 5-FU and its oral products capecitabine and tegafur

5-FU is a cytotoxic agent that belongs to the class of antimetabolites. It is administered intravenously and has a short half-life (10-15 min). 5-FU undergoes a series of bio-transformative reactions that convert it into nucleotide metabolites; one of these metabolites forms a complex with the enzyme thymidylate synthase with inhibition of DNA synthesis through a “thymineless death” mechanism. Capecitabine and tegafur are pro-drugs of 5-FU; they have a bioavailability of approximately 70%-80% after oral administration^[2,3,14]^.

Only a small fraction of 5-FU (1%-5%) is converted into cytotoxic metabolites. More than 80% of the 5-FU undergoes hepatic metabolism by the DPD enzyme whose action consists in the transformation of 5-FU into its inactive product dihydrofluorouracil which is degraded and eliminated through the urine. This causes the DPD to be the rate-limiting step of the inactivation of 5-FU. Other factors such as age, race, comorbidities and concomitant therapies can influence metabolism^[5,13,14]^.

The activity of the DPD presents numerous inter-individual variations. In about 3%-8% of the population there is a partial lack of DPD expression which determines a reduction of the enzymatic activity of about 50%^[15,16]^. Approximately 0.1% has a complete DPD deficit; this implies null enzymatic activity^[10,16-18]^.

In patients that receive 5-FU-based therapy and who have partial DPD deficiency, decreased enzymatic activity results in a reduction in 5-FU inactivation and also in a high risk of severe or fatal toxicity^[15,19]^. Retrospective assessment of patients who developed severe toxicity during treatment with 5-FU, showed that 39%-61% had reduced activity of DPD enzyme^[20-23]^. The DPD is encoded by the *DPYD* gene located on chromosome 1p22 and composed of 23 exons^[24]^. DPD deficiency is usually the result of genetic polymorphisms of *DPYD*. *DPYD* is a highly polymorphic gene; over 50 polymorphic variants have been described^[20,25]^. The screening of the *DPYD* variants is a valuable aid in clinical practice in predicting the occurrence of any toxicity in patients with DPD deficiency^[25,26]^.

## DPD and toxicity

It is confirmed that genetic variation of *DPYD*, that leads to DPD deficiency, can cause severe toxicity in patients treated with a fluoropyrimidine-based chemotherapy. Because of the high doses administered, DPD-deficient patients are likely to experience life-threatening toxicities^[27]^. This pharmacogenetic “DPD syndrome” manifests typically as severe or fatal diarrhea, mucositis/stomatitis, myelosuppression and even rarer toxicities, such as hepatitis and encephalopathy^[28]^. It is hypothesized that safety could be improved by prospective evaluation of DPD-deficiency before treatment start and through adequate dose-adjustment.

Many different methods are used to test DPD deficiency and they can be mainly divided into three groups: tests aimed at assessing DPD enzyme activity, mRNA variants and genetic variants in *DPYD* gene^[29]^.

In the routine the best way to identify DPD-deficient patients is measurement of physiological plasma dihydrouracil/uracil (UH2/U) ratio. Analysis of uracil (U) and dihydrouracil (UH2) is performed in frozen plasma using a high-performance liquid chromatography with ultraviolet detection method; DPD deficiency is reflected by plasma UH2/U decrease or plasma uracil increase. Launay *et al*.^[27,30]^ demonstrated how upfront DPD testing with this ratio and tailored dosing can reduce the incidence of toxicities, while maintaining optimal efficacy in patients treated with 5-FU in a variety of settings.

Due to the difference in terms of enzymatic activity of each variant they will be treated separately in the following section.

### Dose individualization based on specific genotype

Numerous genetic variants in *DPYD* gene are known to change the protein sequence or mRNA splicing process; the consequence is that some of them do not affect DPD activity, instead of other that can cause severe deficiency and related adverse events. Four variants are of primary relevance, because of their frequency in population and their clinical impact (DPYD* 2A, *DPYD* *13, c.2846A>T e c. 1129-5923C>G (HapB3)^[31]^.

DPYD*2A genotype (IVS14+1G>A; c.1905+1G>A; rs3918290) is the first described as being functionally relevant and the most studied polymorphism^[32]^. This allele frequency varies between 0.1% and 1% in African-American and Caucasian population respectively^[33-36]^. Its located at the intron of exon 14 and results in skipping of all the exon and in a nonfunctional protein^[32,37]^.

“*In vitro*” studies showed several *DPYD* variants that were homozygous in mammalian cells; the enzymatic activity of DPD protein was absent when it was assessed in DPYD*2A carriers^[38]^. So, it can be assumed that heterozygous carriers of this variant have approximately half of enzyme activity.

Deenen *et al*.^[26]^ conducted a prospective clinical trial focused on DPYD*2A-guided dosing of fluoropyrimidines, as to prevent severe toxicity. Patients who would be treated with fluoropyrimidine-based chemotherapy were genotyped for DPYD*2A before treatment start: DPYD*2A variant allele carriers were treated with a starting dose reduction of 50%. Then, if well tolerated, the patients would receive a dose-titration phase. Toxicity data for variant allele patients treated with a reduced dose were compared with controls from literature, i.e., DPYD*2A variant allele carriers receiving standard dose, since of the lack of a proper treatment arm with full-dose fluoropyrimidine-based chemotherapy: in particular, due to the already well-known association between DPYD*2A and increased risk of severe and lethal toxicity it was considered unethical to have such a treatment arm.

The study showed that risk of severe (grade ≥ 3) treatment-related toxicity was significantly lower in DPYD*2A variant allele carriers undergoing genotype-guided dosing than in the historical controls, respectively, 28% and 73% (*P* < 0.001). Drug-related death was reduced from 10% in historical controls to 0% in this study.

### Other three variants

c.2846A>T variant allele (D949V; rs67376798) causes a structural change in DPD enzyme that leads to a deficient function^[39]^. Allele frequency varies from 0.1% in African-Americans to 1.1% in Caucasians^[32,39-41]^. *In vitro* data by Offer *et al*.^[41]^ reveal that patients with homozygous expression of this variant have a 59% activity, compared to WT. These results show that even if enzyme activity is significantly reduced it is also more active if compared to homozygous carriers of DPYD*2A^[37]^.

We can deduce that if homozygous expression of c.2846A>T variant allele causes approximately 50% enzyme activity reduction, heterozygous carriers are likely to have 25% reduction in DPD activity; it is then suggested that they would advantage from a 25% drug dose reduction^[34]^.

c.1679T>G (DPYD*13; I560S; rs55886062) is a rare *DPYD* polymorphism: its frequency varies from 0.07% to 0.1% in Caucasians^[32,40]^. *In vitro* study by Offer *et al*.^[37]^ demonstrated that homozygous carriers have a reduction of 75% in DPD enzyme activity and suggests that the heterozygous carriers would have around 50% reduction in DPD enzyme activity.

c.1236G>A polymorphism (E412E; rs556038477) is in exon 11 and it is in complete linkage with all the variants named haplotype B3 (HapB3) (c.483+18G>A, c.680+139G>A, c.959-51T>G and c.1129-5923C>G)^[42,43]^. The c1129-5923C>G intronic polymorphism (rs75017182) located deep in intron 10 causes abnormal splicing and maybe it is responsible for the DPD enzyme deficiency^[31,43]^.

The frequency of heterozygous patients in Caucasian populations varies between 2.6% and 6.3%^[42-46]^. DPD activity is not completely absent in homozygous carriers; so it is expected that a 25% dose reduction for heterozygous carriers is convenient^[34,47]^.

The clinical validity of all these three variants, in particular the toxicity related to fluoropyrimidine-based chemotherapy in patients carriers of these mutations, was studied by Meulendijks *et al*.^[48]^ in a systematic review and meta-analysis. They collected data from 7356 patients and demonstrated that c.1679T>G, c.1236G>A, and c.2846A>T are clinically relevant predictors of fluoropyrimidine-associated toxicity, as the DPYD*2A variant.

On the base of these findings, it can be expected that initial dose reductions in heterozygous carriers of these three other *DPYD* polymorphisms will result in reduction of toxicity^[49]^ [Table 1].

**Table 1:** Dose-reduction based on DPYD variant

DPYD variant	% of standard fluoropyrimidine dose
DPYD*2A (rs3918290)	50%
c.1679T>G (rs55886062)	50%
c.2846A>T (rs67376798)	75%
c.1236G>A/HapB3 (rs56038477)	75%

Henricks *et al*.^[50]^ published a multicenter prospective study to investigate the effect of dose-individualization chemotherapy, on the basis of four *DPYD* variants, on fluoropyrimidine drugs toxicity. Patients were genotyped for the four variants of *DPYD* and received an initial dose reduction of 5-FU following the current guidelines^[31]^. This study shows that dose-individualization is achievable and can improve patient safety: it reduces risk of severe toxicity for DPYD*2A carriers, it is safe in c.1679T>G variant and decrease the toxicity risk in c.2846A>T, although the risk was still higher than WT. For c.1236G>A carriers, a reduction of 25% is not enough to diminishing toxicity. When compared with patients in historical cohort, the frequency of severe toxicity in DPYD*2A carriers was lower (31% *vs*. 72%); the only patient with c.1679T>G did not have toxicity after 50% dose reduction (the authors could not make correlation because of it was only one). For c.1236G>A and c.2846A>T reduction of 25% seemed not safe: 39% of the first group and 47% of the second had severe toxicity. The authors proved even that dose adjustment chemotherapy can reduces hospital admission due to severe toxicity and therapy discontinuation of treatment. The authors compared the patients treated with genotyped-dose reduction RR for severe toxicity with the same RR in *DPYD* variant allele carriers from a historical cohort from a meta-analysis, (in this last study, patients were not genotyped before treatment and received a full dose chemotherapy)^[48]^.

## Impact of DPD expression in different cancer types

### Colorectal cancer

The role of DPD expression as a predictor of effectiveness of 5-FU has been highly investigated in CRC, in comparison to other types of cancer. However, definitive consensus on its reliability as predictive factor has not been reached yet. Below the studies in the metastatic and adjuvant setting are disussed separately given the difference between advanced and localized disease in terms of prognosis and biology.

Some studies analyzed the level of response of advanced CRC to 5-FU chemotherapy in relation to DPD values. Vallböhmer *et al*.^[51]^ demonstrated a statistically significant correlation between DPD expression and response to 5-FU based chemotherapy. This study enrolled 37 patients with metastatic CRC treated with first-line capecitabine whose intratumoral DPD mRNA levels were assessed using laser capture microdissection and real-time (RT) PCR. Patients that had at least 50% tumor shrinkage at the CT scan were classified as responders to therapy. The results highlighted a significant association between high levels of intratumoral DPD and resistance to capecitabine while patients with lower mRNA expression levels of DPD showed better progression free survival (PFS).

A correlation between intratumoral gene expression levels of DPD and response to 5-FU was also reported by a study conducted by Salonga *et al*.^[52]^. By using a pre-established cut-off value (DPD: b-actin, 2.5 × 10³), authors confirmed the negative predictive value of high levels of DPD (patients who have higher levels of DPD show actually no response after 5-FU based therapy compared with patients with lower levels of DPD where a response rate of 50% was seen).

In addition to studies that suggested a potential correlation between DPD expression and 5-FU-based chemotherapy activity, there are a few reports where this assumption was not proven, either in patients with metastatic or unresectable locally advanced colorectal cancer^[53,54]^.

Other studies have assessed the role of DPD expression values as a prognostic factor in metastatic CRC. A large Phase III study conducted by Koopman *et al*.^[55]^ (CAIRO) randomized 803 advanced CRC patients between sequential treatment (first-line: capecitabine, second-line: irinotecan, third-line: capecitabine plus oxaliplatin) or combination treatment (first-line capecitabine plus irinotecan, second-line: capecitabine plus oxliplatin). Retrospective evaluation of DPD expression by immunohistochemistry (IHC) was performed in formalin-fixed paraffin-embedded samples obtained from 556 patients. A statistically significant positive predictive value for low versus high DPD values was noted in 283 patients treated with capecitabine plus irinotecan, in terms of improved median PFS and overall survival (OS)^[55]^.

Yanagisawa *et al*.^[53]^ also highlighted a positive correlation of low DPD expression and OS in a small number of patients affected metastatic CRC treated with a regimen composed by 5-FU, irinotecan and leucovorin (MIFL). Further studies evidenced a benefit in terms of PFS^[51]^ and time to progression^[56]^ in patients with low DPD expression treated respectively with capecitabine and first-line 5-FU based regimen.

The potential influence of the DPD expression on outcome in patients treated with 5-FU based chemotherapy has been also investigated in adjuvant setting.

Ciaparrone *et al*.^[57]^ investigated the prognostic role of DPD values (evaluated by IHC) in 62 patients who underwent surgery and received 5-FU adjuvant treatment. At multivariate analysis, high DPD expression was significantly correlated with worse DFS and OS. Further studies reported a shorter DFS^[58-60]^ and both worse DFS and OS^[61]^ in patients with high DPD expression. Some studies evidenced that low DPD expression levels were associated with trends for better OS^[60,62]^ even if not statistically significant. Kornmann *et al*.^[63]^ examined the prognostic value of thymidylate synthase (TS) and DPD expression in 295 patients who received adjuvant 5-FU based chemotherapy after surgery. Patients who had tumors with low TS and high DPD levels had worse prognosis in contrast with the ones who expressed high TS and low DPD.

Nevertheless, some other studies, reported no correlation between DPD expression and prognosis in this setting^[64-66]^. These results taken together seem to suggest a correlation between low DPD expression and better prognosis in patients with colorectal cancer treated with 5-FU based chemotherapy in both metastatic [Table 2] and adjuvant setting [Table 3]. However, the retrospective nature of these trials, the small number of patients enrolled, the use of different chemotherapy regimens and the presence of conflicting data, question the reliability of DPD expression as a predictive factor in this context. Smorenburg *et al*.^[67]^ realized the only prospective study in which the expression of TS and DPD (in 53 patients with advanced colorectal cancer), led the choice of the first line chemotherapy. They proved that patients who had low TS and low DPD levels have higher response rate to 5-FU/LV compared with patients treated with non 5-FU containing regimen. The most relevant limitation of this study is that the population was divided into two groups with different markers and treated with different chemotherapy; so this does not allow a comparison between the groups and does not confirm the DPD expression predictive role.

**Table 2:** Dihydropyrimidine dehydrogenase expression correlation with response and outcome in patients with advanced colorectal cancer receiving fluoropyrimidine-based chemotherapy

Study (ref.)	Treatment setting	Regimen	Patients (*n*)	Method	Better response	Longer TTP/PFS	Longer OS
Salonga *et al*.^[52]^	Advanced	5-FU + LV	33	RT-PCR	Low DPD exp.		
Meropol *et al*.^[54]^	Advanced	CAPIRI	67	RT-PCR	NS		
Vallböhmer *et al*.^[51]^	Advanced	CAP-based	37	RT-PCR	Low DPD exp.	Low DPD exp.	
Yanagisawa *et al*.^[53]^	Advanced	5-FU + LV	21	RT-PCR	NS		Low DPD exp.
Gustavsson *et al*.^[56]^	Advanced	5-FU-based	144	RT-PCR		Low DPD exp.	
Koopman *et al*.^[55]^	Advanced	CAPIRI	283	IHC		Low DPD exp.	Low DPD exp.

CAP: capecitabine; CAPIRI: capecitabine + irinotecan; DPD: dihydropyrimidine dehydrogenase; ELISA: enzyme-linked; Exp: expression; IHC: immununohistochemical analysis; LV: leucovorin; NS: not significant; OS: overall survival; PFS: progression-free survival; RT-PCR: reverse transcriptase PCR; TTP: time to progression; 5-FU: 5-fluorouracil

**Table 3:** Dihydropyrimidine dehydrogenase expression correlation with response and outcome in patients with resected colorectal cancer receiving fluoropyrimidine-based chemotherapy

Study (ref.)	Treatment setting	Regimen	Patients (*n*)	Method	Better response	Longer DFS	Longer OS
Kornmann *et al*.^[65]^	Adjuvant	5-fu-based	348	RT-PCR		NS	NS
Ikeguchi *et al*.^[66]^	Adjuvant	5-FU	100	ELISA			NS
Kornmann *et al*.^[63]^	Adjuvant	5-FU-based	295	RT-PCR			Low DPD exp.
Oi *et al*.^[58]^	Adjuvant	5-FU-based	64	IHC		Low DPD exp.	
Westra *et al*.^[64]^	Adjuvant	5-FU-based	341	IHC		-	
Ciaparrone *et al*.^[57]^	Adjuvant	5-FU + LV	62	IHC		Low DPD exp.	Low DPD exp.
Lassman *et al*.^[59]^	Adjuvant	5-FU-based	52	RT-PCR		Low DPD exp.	
Hotta *et al*.^[60]^	Adjuvant	5-FU + LV	22	ELISA		Low DPD exp.	NS
Jensen *et al*.^[61]^	Adjuvant	5-FU + IV	303	IHC		Low DPD exp.	Low DPD exp.
Soong *et al*.^[62]^	Adjuvant	5-FU + LV	123	IHC			NS

DFS: disease-free survival; DPD: dihydropyrimidine dehydrogenase; ELISA: enzyme-linked immunosorbed assay; Exp: expression; IHC: immununohistochemical analysis; IV: isovorin; LV: leucovorin; NS: not significant; OS: overall survival; RT-PCR: reverse transcriptase PCR; 5-FU: 5- fluorouracil; -: no positive correlation

The B-CAST study is a multicenter, prospective cohort trial aimed to identify a correlation between tumor biomarkers expression, including DPD expression determined by RT-PCR, and outcome benefit from adjuvant treatment with different 5-FU based regimens in a population with stage III colon cancer: preliminary results of the study are actually unavailable, but we believe that this study might contribute to clarify the role of DPD as a predictive marker in this setting, owing to the large number of patients (2128) enrolled.

### Gastric cancer

5-FU based chemotherapy is commonly used for both palliative treatment of metastatic gastric cancer (GC) and adjuvant therapy in patients who underwent surgery. Therefore the role of DPD expression as a predictive biomarker for chemotherapy sensitivity and for outcome in patients treated with 5-FU has been widely investigated in this setting.

Toriumi *et al*.^[68]^ reported a statistically significant correlation, *in vitro*, between sensitivity to 5-FU therapy and DPD expression: in particular, cells that exhibited high expression of DPD mRNA were resistant to 5-FU; a weak inverse correlation between DPD level and sensitivity to 5’-deoxy-5-ﬂuorouridine (5’-DFUR) *in vitro* was also reported by Terashima *et al*.^[69]^. Some other studies also showed high DPD expressions levels in 5-FU resistant cell lines^[70,71]^ while DPD mRNA expression did not correlate with 5-FU sensitivity in a study conducted by Kodera *et al*.^[72]^. *In vivo* studies by Nishina *et al*.^[73]^ and by Koizumi *et al*.^[74]^, conducted in a population affected by advanced GC, suggested that patients with high thymidine phosphorylase (TP) and low DPD expression are more sensitive to 5’-DFUR^[73]^ and capecitabine^[74]^ respectively. However a low DPD association has been associated with worst response rate in patients treated with S-1^[75]^, a chemotherapy containing 5-chloro-2,4-dihydroxypiridine (CDHP) with anti-DPD activity widely used in Japan, and other studies demonstrated not statistically significant results with the same agent^[76]^.

Similarly to colon cancer patients, also in GC, the predictive role of DPD was investigated in both metastatic and adjuvant setting.

In adjuvant setting a longer survival was reported in patients treated with 5’-DFUR chemotherapy showing high TP to DPD ratio^[69]^. Opposite results were reached by other two studies, where low intratumoral DPD expression was correlated with worse DFS^[77]^ and OS^[78]^ in patients who received S-1 adjuvant therapy.

A study conducted by Grau *et al*.^[79]^ investigated the role of genetic variations in *DPYD* in place of looking at DPD expression, analyzing the prognostic value of genetic single nucleotide polymorphisms (SNPs) of intratumoral *DPYD* and CDA (cytidine-deaminase enzyme, which can influence the activity of TS) in patients with GC treated with adjuvant fluoropyrimidine (tegafur). SNP of *DPYD*1 (A/G; Ile453Val) was associated with better survival while the SNP (C/T; Arg29Cys) of *DPYD*2 showed a benefit in terms of relapse and survival if associated with the polymorphism of CDA (A/C; Lys27Gln)^[79]^.

Conflicting results are reported also in the metastatic setting. Some studies have demonstrated that high DPD expression can be a predictor of poor survival in patients treated with S-1^[80,81]^ and 5-FU^[68]^, while a study conducted by Nishina *et al*.^[73]^ highlighted a connection between high TP to DPD ratio and longer OS in patients treated with 5-DFUR chemotherapy. At the same time various studies reported the absence of statistically significant impact of DPD value on survival in patients treated with S-1^[75,76,82]^ and 5-FU^[75,83]^.

Given the controversial results previously reported, in 2017, Zhang *et al*.^[84]^ performed a meta-analysis aimed to assess the potential impact of intratumoral DPD expression level on chemotherapy sensitivity and long-term survival for GC. The meta-analysis confirmed a correlation between high DPD expression and 5-FU activity, whereas long-term survival was not significantly different.

It should be noted that, while studies that assessed the activity of the chemotherapy regimens were conducted mainly by using 5-FU, studies that contributed in the meta-analysis to survival outcomes were those where S-1 was used more frequently. S-1 is a combined drug consisting of tegafur and CDHP, a chemotherapeutic agent characterized by DPD inhibitor activity; proof of this activity is the absence of hand-foot syndrome due to the lower concentration of 5-FU degradation products in patients treated with S-1 unlike patients treated with 5-FU or capecitabine. Thus, an enhancement of the antitumor effect would be expected in patients with high intratumoral DPD expression in comparison with patients treated with 5-FU, potentially invalidating the prognostic role of DPD expression level. While part of the previously cited studies confirm this hypothesis highlighting no significant prognostic difference between patients with high or low DPD expression or a worse prognosis in case of patients with low DPD levels treated with S-1, other studies provide opposite results. In fact, even if CDHP inhibiting activity *in vitro* is established, the degree of intratumoral DPD activity inhibited by this agent in clinical setting is unknown.

Furthermore, the retrospective design of these studies, coupled with the lack of a reproducible assay for DPD expression, represents a severe limitation that could explain the lack of consistent data. Moreover, due to the fact that the majority of the studies included in the meta-analysis were conducted in Japan, due to the aforementioned described ethnic differences in terms of DPD expression, definitive conclusions concerning the association between DPD and sensitivity to chemotherapy can not apply to all populations.

### Other cancer types

The role of DPD expression as a prognostic factor has been mainly investigated in CRC and GC as previously described, however the widely use of 5-FU based chemotherapy has encouraged researchers to examine its value as a predictive and prognostic factor in other tumor types. Some studies showed a survival benefit in patients with low DPD expression treated with adjuvant S-1^[85]^ or 5-FU^[86,87]^ after the resection of pancreatic cancer. However, in a study conducted by Murakawa *et al*.^[88]^, no significant difference in terms of 3-year OS following surgery was reported between DPD-postive and DPD-negative expression groups in patients treated with S-1 chemotherapy. In other studies which enrolled a small number of pancreatic cancer patients adjuvantly treated with 5-FU based chemotherapy, low TP/DPD ratio was significantly correlated with longer survival^[89,90]^.

In regard to head and neck cancer, intratumoral overexpression of DPD has been correlated with resistance to 5-FU-based chemotherapy in metastatic^[91]^ and neoadjuvant setting^[92]^. In contrast, in a recent study conducted by Hasegawa *et al*.^[93]^, higher DPD expression was predictive of better response to induction chemotherapy with 5-FU/cisplatin.

Few studies has been carried out in breast cancer patients treated with FU-based treatment. While some studies assessed a role of low DPD expression as a predictive factor for chemosensitivity^[94]^ and for DFS in adjuvant setting^[95]^, other ones did not found a significant difference between high and low DPD groups in terms of DFS in adjuvantly treated patients^[96,97]^ and OS in metastatic ones^[98]^. A recent study conducted by Qin *et al*.^[99]^ was the first aimed to investigate the prognostic role of *DPYD* polymorphisms in breast cancer. They demonstrated that *DPYD* c.1627A>G AG/GG polymorphism, detected from tumor tissue, was associated with poor OS and PFS in non-luminal patients treated with fluoropyrimidine-based chemotherapy^[99]^.

Conflicting data are also reported in lung cancer patients, with DPD expression pointed out as either related^[100]^ or not significantly associated with prognosis^[101]^.

In addition to the described limitations of the studies regarding the potential predictive and prognostic role of DPD expression in CRC and GC, the relative small number of investigations focused on different tumor types makes a hypothetical consensus unreachable at the moment.

## CPIC^®^ and dose adjustment

Clinical Pharmacogenetics Implementation Consortium (CPIC^®^) is an International Consortium establishing guidelines interested in facilitating use of pharmacogenetic tests for patient care; it helps clinicians interpreting genetic test results and ensuring a safer drug delivery to the patient^[102]^.

In the CPIC^®^ Guidelines for *DPYD* genotype and fluoropyrimidine dosing it is summarized the relationship between *DPYD* polymorphism deficiency and the dose-adjustment of 5-FU based chemotherapy.

In the 2013 edition, initial dose reduction was recommended only for the c.2846A>T, c.1679T>G and DPYD*2A polymorphisms; if after two chemotherapy cycles the treatment proved to be safe, individual dose can be titrated to have maximum safe drug concentration in every patient^[49]^.

In the 2017 update all the four *DPYD* variants discussed above have been identified as relevant due to their frequency, their impact on enzyme function and clinical toxicity. Among them, DPYD*2A and DPYD*13 have the most dangerous consequences on DPD enzyme activity; c.2846A>T and c.1129-5923C>G variants cause a moderate activity reduction.

The most common decreased function DPD variant in Europe is HapB3 with c.1129-5923C>G (4.7%), followed by c.1905+1G>A (1.6%) and c.2846A>T (0.7%). Adding all the four variants 7% of Europeans carry one decreased function *DPYD* allele. All the other allele frequency are rarer^[31]^.

In Table 4 are shown correlations between DPD phenotype and *DPYD* genotype, assessed only for few variants, because the functional impact of rarer polymorphism has been only evaluated *in vitro*. The DPD phenotype is assigned using a gene activity-score (*DPYD*-AS): carriers of two no functional alleles are classified as *DPYD* poor metabolizers (*DPYD*-AS:0); carriers of one no function or decreased function are *DPYD* intermediate metabolizers (*DPYD*-AS:1 or 1.5) and those with normal function alleles are *DPYD* normal metabolizers (*DPYD*-AS:2).

**Table 4:** Assignment of likely DPD phenotypes based on *DPYD* genotypes

Likely phenotype	Activity score	Genotypes	Examples of Genotypes
Normal metabolizer	2	Carriers of two normal function alleles	c.[ = ];[ = ], c.[85T>C];[ =], c.[1627A>G];[ = ]
Intermediate metabolizer	1 or 1.5	Carriers of one normal function allele plus one no function allele or one decreased function allele, or carriers of two decreased function alleles	c.[190511G>A];[ = ], c.[1679T>G];[ = ], c.[2846A>T];[ = ]; c.[1129-5923C>G]; [ = ]; c.[1129-5923C>G];[1129-5923C>G]; c.[2846A>T];[2846A>T]
Poor metabolizer	0 or 0.5	Carriers of two no function alleles or carriers of one no function plus one decreased function allele	c.[190511G>A];[190511G>A], c.[1679T>G];[1679T>G], c.[190511G>A];[2846A>T] c.[190511G>A]; [1129-5923C>G]

Table 5 contains the genetic-based recommendations for fluoropyrimidines-dose using the calculated *DPYD* activity score (*DPYD*-AS).

**Table 5:** Recommended dosing of Fluoropyrimidines by *DPD* phenotype

Phenotype	Clinical consequences	Dosing recommendations	Classification of recommendations
*DPYD* normal metabolizer	Normal DPD activity.	Label-recommended dosage and administration.	Strong
*DPYD* intermediate metabolizer	Decreased DPD activity and increased risk for drug toxicity.	Reduce starting dose based on activity score followed by titration of dose based on toxicity or therapeutic drug monitoring (if available). Activity score 1: Reduce dose by 50% Activity score 1.5: Reduce dose by 25% to 50%	Activity score 1: strong Activity score 1.5: moderate
*DPYD* poor metabolizer	Complete DPD deficiency and increased risk for drug toxicity	Activity score 0.5:avoid use of fluoropyrimidines. If there are not alternatives agents, 5-FU should be administered at a strongly reduced dose. Activity score 0: Avoid fluoropyrimidines	Strong

Patients heterozygous for *DPYD* decreased/no function variants should receive reduced starting dose because of partial enzyme activity. In particular heterozygous carriers of DPYD*2A treated with 50% dose reduction showed a rate of severe toxicity comparable to non carriers (*DPYD*-AS:1)^[38]^. In patients with c. 2846A>T heterozygous polymorphism a retrospective study established that the capecitabine starting dose was reduced by 25% compared to non carriers^[44]^, just like the carriers of c.1236G>A^[103]^. So the finally deduction is that patients carriers of DYD-AS:1.5 can tolerate higher doses than patients carriers of no function variants (*DPYD*-AS:1). In patients with decreasing function other circumstance should be considered to determine the initial dose-reduction, 50% starting dose followed by dose titration or only a 25% dose reduction.

If the first two cycles are well tolerated, to maintain effectiveness dose could be increased in subsequent cycles, given that some patients, even if carriers of no function or less function variants, tolerate normal dose of 5-FU. Similarly, if no tolerated, dose should be decreased.

In *DPYD* poor metabolizers (*DPYD*-AS: 0.5 or 0) it is strongly recommended to avoid 5-FU based chemotherapy. However, if there are no other options, 5-FU regimen at a heavily reduced dose in association with early drug monitoring may be considered for *DPYD*-AS:0.5. It should be noted that no reports of successful administration of a low dose of 5-FU in DPD poor metabolizers are available. It is estimated that a dose-reduction of 75% would be required. The CPIC defines as poor metabolizer only patients with two no-functional alleles; this score could be considered poor sensitive and high specific. We speculate that this approach aims to avoid false positive results in order not to deprive patients of 5-FU, given its primary role in solid tumors treatment.

## DPD and costs

*DPYD* mutations tests are not routinely performed because of the initial costing concerns of the tests and the absence of clear dose reduction guidelines in patients who have a deficit of DPD in prospective tests^[104]^. Considering the ever-increasing number of patients that will require fluoropyrimidine-based chemotherapy, concerns regarding also the usefulness and costs of prospective testing have arisen.

Furthermore, the absence of a mutation does not guarantee the absence of serious toxicity. Conversely, the potential benefit of prospective identification of *DPYD* mutations is that, careful monitoring and gradual dose escalation, may allow patients with DPD deficiency to receive fluoropyrimidine chemotherapy safely^[6,105]^.

Murphy *et al*.^[106]^ have shown that the cost of managing patients with severe toxicity to chemotherapy and with *DPYD* reactive test is higher than the cost of the prospective test on each new patient who initiates fluoropyrimidine chemotherapy. These results therefore showed that tests for the detection of *DPYD* mutations in the study population would be associated with significant cost savings. Another not-randomized study^[107]^ showed that prospective tests for DPD deficiency in patients receiving 5FU-based chemotherapy for CRC could be a cost-effective strategy.

Prospective identification of patients with a *DPYD* mutation, associated with a dose reduction from the start of therapy, can avoid chemotherapy-related toxicity and improve quality of life. In clinical practice, it is therefore reasonable to reduce the doses appropriately for the best characterized polymorphisms, avoiding testing polymorphisms with a still undefined meaning. In patients with these polymorphisms, greater vigilance may be suggested without dose reductions from the start of therapy. Routine prospective tests are therefore economically viable even if further research and clear dose reduction recommendations are needed. The *DPYD* test has the potential to prevent premature cessation of potentially curative therapy for patients with deficiencies^[106,107]^.

## Conclusions

Fluoropyrimidines still remain a class of pivotal drugs in the treatment of solid tumors. In this context the DPD enzyme plays an essential role as responsible for the inactivation of 5-FU. To date, many polymorphisms of the gene coding for this enzyme are known, determining various degrees of toxicity in patients who are treated with these drugs. The studies focused on 4 of these genotypes, those with the highest reported frequency in the population and those with a greater clinical impact: DPYD*2A, c.1679T>G, c.2846A>T and c.1236G>A. These polymorphisms differ in the degree of toxicity and therefore each patient should receive dosage of these drugs that is based on the activity of this enzyme.

Furthermore, the expression of DPD should have profound impact in terms of prognosis and as predictor of effectiveness of these drugs. Speaking from a prognostic point of view, the presence of high intratumoral DPD expression should be associated with chemoresistance and worst outcome. The validation of this assumption would guide clinicians in everyday practice, encouraging, for instance, the use of 5-FU-based adjuvant therapy in patients at intermediate risk of recurrence after surgical resection or, moreover, the use of fluoropyrimidine-including treatment in metastatic setting, in presence of low DPD expression. On other hand, from a predictive point of view, due to the paramount importance of 5-FU-based chemotherapy in CRC, low DPD expression should be associated to better response/outcome in patients treated with fluoropyrimidine. This matter has been assessed in a series of studies in the setting of CRC. However, these studies are affected by various limitations: most of these studies are retrospective analyses and the majority of them is characterized by small sample size. We must mention also the lack of a standardized method of measurement of DPD, as it has been carried out in various papers through different ways (RT-PCR, IHC or ELISA), and the absence of a defined cut-off which could differentiate high from low DPD expression. Furthermore, there was no standardization even for chemotherapy regimens, given the use of different types and doses of fluoropyrimidine and the combinations with different chemotherapeutic agents such as cisplatin and oxaliplatin, which could have highly influenced reported results. Therefore, even if there seems to be a trend supporting the correlation between DPD expression and response/survival in patients treated with fluoropyrimidine, no definitive conclusions can be pointed out, considering also that some studies showed not statistically significant or even opposite results. The same statements could be referred to GC setting, which is also affected by limitations of ethnicity (almost all studies were conducted in Japan) and possible results distorsions due to the wide use of S-1 (in Japanese population), whose DPD inhibiting activity may invalidate possible statement concerning the role of DPD expression as a predictive factor.

Thus the debate on the intratumoral DPD expression as a potential predictive and prognostic marker in fluoropyrimidine treated patients, is still open and it is crucial to analyze its role through prospective studies^[108]^, standardized for DPD expression assessment method and chemoterapeutic regimen used. Moreover, given the complexity of 5-FU pharmacokinetic, it would be useful to integrate future prospective studies with the evaluation of the other enzymes involved in 5-FU metabolic pathway.

## Declarations

### Copyright

© The Author(s) 2019.
